# Differentiation of AFP-negative hepatocellular carcinoma from other intrahepatic malignant lesions by a noninvasive predictive model based on Sonazoid contrast-enhanced ultrasound

**DOI:** 10.3389/fonc.2025.1623670

**Published:** 2025-07-17

**Authors:** Qian Zhang, Zhilong Liu, Ruining Wang, Lele Song, Wenwen Fan, Ping Liang, Liping Liu

**Affiliations:** ^1^ Department of Interventional Ultrasound, First Hospital of Shanxi Medical University, Taiyuan, Shanxi, China; ^2^ Department of Interventional Ultrasound, Fifth Medical Center of Chinese PLA General Hospital, Beijing, China

**Keywords:** AFP-negative, hepatocellular carcinoma, contrast-enhanced ultrasound, Sonazoid, nomogram

## Abstract

**Objectives:**

This study aimed to develop and validate a non-invasive predictive model, which was a reliable nomogram to accurately differentiate AFPN-HCC from other intrahepatic malignant lesions.

**Methods:**

This study enrolled 165 patients with malignant focal liver lesions, including AFPN-HCC (n=85) and other intrahepatic malignant lesions (n=80). Data were analyzed to screen for risk factors phase by using LASSO regression as well as univariate and multivariate logistic regression analysis. We constructed a model and developed a nomogram. Then using the area under the curve, Hosmer-Lemeshow test, calibration curves, decision curve analysis, and 1,000 bootstraps to assess and internally validate the model performance. We calculated the optimal threshold, sensitivity, specificity, positive and negative predictive value, and accuracy of the prediction model.

**Results:**

LASSO and multivariate logistic regression analyses indicated that tumor number, necrosis in tumor, arterial phase enhancement pattern, arterial phase perfusion velocity, and Kupffer phase degree of washout were the significant predictors to differentiate AFPN-HCC from OM. The AUC was 0.886, and the AUC of internal validation was 0.865. The optimal critical value of the predicted value was 0.524, with a sensitivity of 82.35%, specificity of 85.00%, positive predicted value of 85.37%, negative predicted value of 81.93%, and an accuracy of 83.64%. The *P* value of the Hosmer-Lemeshow test was 0.592. The calibration plots showed a high concordance of prediction. The decision curve analysis showed excellent net benefits.

**Conclusion:**

Our nomogram has excellent discrimination, calibration and clinical utility by combining SCEUS and clinical features, which may help clinicians improve the diagnostic performance for AFPN-HCC, contributing to individualized treatment.

## 1 Introduction

Hepatocellular Carcinoma (HCC) accounts for 75%-85% of primary liver cancers, representing the sixth most prevalent cancer and the third leading cause of cancer-related mortality around the world (1, 2). HCC has caused 800,000 deaths worldwide, threatening human health and representing a major global healthcare challenge (3). What’s more, HCC has a lower 5-year survival rate of 18%, and a higher 5-year recurrence rate of more than 70% (4). Therefore, early detection and timely intervention is very important.

Alpha-fetoprotein (AFP) is clinically used as the serological marker for the diagnosis of HCC, but about 30-40% of patients with HCC have normal serum AFP levels (< 20 ng/mL), which is known as AFP-negative HCC (AFPN-HCC) (5). It is easily to misdiagnose AFPN-HCC patients as other intrahepatic malignant lesions (OM), but the treatment modalities for them are not identical. Thus, early and accurate preoperative diagnosis and differentiation of AFPN-HCC from OM are especially essential for the clinical treatment options and prognosis optimization.

The early diagnosis of AFPN-HCC relies on imaging, but conventional ultrasound is susceptible to interference from the background of liver cirrhosis, and has similar features with intrahepatic cholangiocarcinoma (ICC) and hepatic metastases, which poses a challenge to the clinical differential diagnosis. Contrast-enhanced ultrasound (CEUS) can provide finer hemodynamic information by real-time dynamically observing tumor microcirculatory perfusion. Sonazoid contrast-enhanced ultrasound (SCEUS) provides additional diagnostic information for liver lesions due to the advantages of long image enhancement time and unique Kupffer phase (KP), thus it can significantly improve the detection accuracy of liver tumors (6).

Current studies focus on the diagnostic efficacy of CEUS LI-RADS classification on HCC, with less attention to AFPN-HCC. In this study, we analyzed the characteristics of SCEUS of AFPN-HCC to explore the key points of differentiation between AFPN-HCC and OM, established a preoperative noninvasive prediction model in order to provide a reliable basis for clinical differential diagnosis and treatment.

## 2 Methods

### 2.1 Participants

The study was approved by the Ethics Committee of the First Hospital of Shanxi Medical University (No. KYLL 2023-132) and complied with the Declaration of Helsinki. All enrolled patients signed an informed consent form.

**Figure 1:**
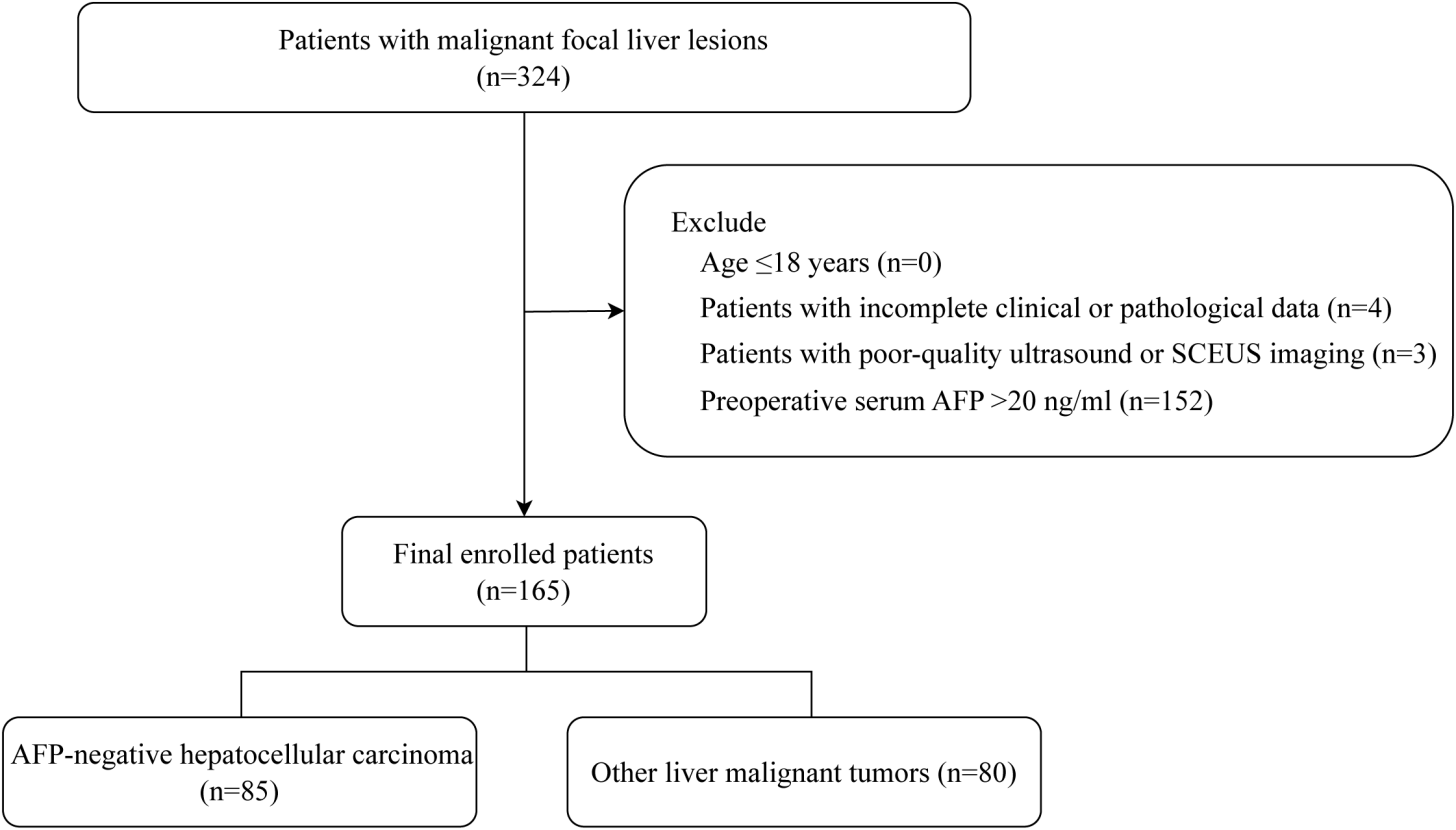
Flowchart shows selection criteria for the 165 patients in the study group.

We collected patients with malignant focal liver lesions who underwent SCEUS before surgery from September 2020 to December 2024 (n=324). The inclusion criteria were as follows: (1) age > 18 years; (2) liver function classified as Child-Pugh class A; (3) no local or systemic treatment was received before the examination; (4) no allergies to any components of Sonazoid; (5) voluntary participation and signed informed consent; (6) a definitive pathological diagnosis by puncture or surgery; (7) preoperative serum AFP ≤ 20 ng/ml. Exclusion criteria were: (1) age ≤ 18 years; (2) patients with incomplete clinical or pathological data; (3) patients with poor-quality ultrasound or SCEUS imaging (4) preoperative serum AFP > 20 ng/ml. We finally enrolled 165 participants as the study population. The flow chart of patient enrollment is shown in **Figure 1**.

### 2.2 Baseline clinical data

We collected the clinical data of 165 individuals diagnosed with liver malignant focal lesions, including three parts: demographic characteristics (such as age, gender, BMI), clinical characteristics (such as hypertension, diabetes mellitus), and serum laboratory information (such as fasting plasma glucose, alanine aminotransferase, aspartate aminotransferase, albumin, total bilirubin, gamma-glutamyl transferase, total cholesterol, triglyceride, high-density lipoprotein, low-density lipoprotein, and serum uric acid).

### 2.3 Ultrasound examination

Ultrasound examinations were performed with the Resona R9 (Mindray, Shenzhen, China), equipped with a convex array broadband probe (SC6-1U). The patients took horizontal or left lateral position, with the abdomen exposed and the right arm raised to fully expose the liver area, scanned the liver. Then we observed and recorded the tumor information. Once the probe position was stabilized, the CEUS mode was switched, and the resulting images were visualized in a dual-screen format. Real-time dynamic imaging was carried out using the Sonazoid (GE, Boston, USA) with a mechanical index (MI) of 0.183. Sonazoid (0.01 ml/kg) was injected via the median elbow vein by croup injection, followed immediately by 5ml of saline, carefully timed and recorded continuously for 3 min. Dynamic images were then saved for 10s at 1 min intervals until 12 min after the contrast agent was injected. All these images were saved on the hard drive for follow-up analysis. Vascular and post-vascular phases in CEUS of the liver (visualization post-injection time) (7) were arterial phase (AP), 10-30s; portal venous phase (PVP), 30-120s; delay phase (DP), 2-10min; and KP, starting at 10 min.

The following conventional ultrasound features were gleaned: tumor location, echo, size, number, boundary, morphology and envelope. Meanwhile, the following SCEUS features were also collected: (1) necrosis in tumor, defined as the presence of non-enhancing areas inside the tumor in AP phase; (2) AP enhancement pattern, defined as rim-enhancement and overall-enhancement; (3) AP perfusion velocity, defined as quick or simultaneous enter; (4) AP enhanced level, defined as hyper-, iso- and hypo-enhancement; (5) AP enhanced homogeneity, defined as homogeneous and heterogeneous; (6) AP enhanced margin, defined as well- or poorly defined; (7) AP tumor morphology, defined as regular or irregular; (8) PVP and DP clearance velocity, defined as quick or simultaneous washout; (9) PVP and DP enhanced level, defined as hypo-, iso- and hyper-enhancement; (10) KP enhanced level, defined as hypo- or iso-enhancement; (11) KP degree of washout, with almost no contrast agent retention in the tumor defined as obvious washout, otherwise, it was mild/moderate washout.

Data were completed independently by two researchers with 5 years of experience in abdominal radiology, particularly in liver imaging. If disagreements occurred, the images were assessed by a third radiologist with 30 years of work experience.

### 2.4 Sample size

In the process of developing multivariate predictive models, the sample size is typically based on the proportion of the number of individuals of the outcome event to the number of candidate predictors, known as the events per variable (EPV). According to an empirical study, a thumb rule of at least 10 EPV was proposed and is widely accepted as a means to prevent over-fitting (8). Therefore, we could consider a maximum of 8 variables (85 outcome events/10 EPV). All the available patients were included in this study.

### 2.5 Statistical analyses

All statistical tests were performed using the *R* statistical software (version 4.2.2) and the *Free Statistics* software (version 2.1), with pathological results as the gold standard. A two-sided test *P* value of less than 0.05 was considered significant.

Continuous variables with normal distribution were presented as means ± SD and analyzed by t-test. Non-normally distributed continuous variables were expressed as median and inter-quartile range and analyzed using the Mann-Whitney U test. Categorical factors are described as frequencies or proportions, and analyzed by χ^2^ test. Inter-observer variability analysis was performed using kappa (κ) statistic, as detailed in the **Supplementary Table 1**. The agreement was classified as follows: poor for 0-0.2, fair for 0.2-0.4, moderate for 0.4-0.6, good for 0.6-0.8, and excellent for values greater than 0.8.

We used a robust high-dimensional prediction approach, the least absolute shrinkage and selection operator (LASSO) regression, to ascertain potential predictor variables for AFPN-HCC. Five-fold cross-validation was used to determine the best value of λ. We chose Lambda=1se to determine the final candidate characteristics for the cross-validation results. The features selected in the LASSO regression model were used in univariate and multivariate logistic regression analysis to identify statistically significant predictors, which were then employed in the development of nomogram.

We used the receiver operating characteristic curve and area under the curve (AUC) for assessing the differentiation of the nomogram, and the Hosmer-Lemeshow test for evaluating the calibration of the nomogram. Then we determined the optimal cutoff point for predicting nomogram by maximizing the Youden index, and calculated its sensitivity, specificity, accuracy, positive predictive value, and negative predictive value. We employed bootstrap method for internal validation, in which 1000 samples were randomly selected from the original data for bootstrap replication. Finally, We calculated calibration AUC values and plotted calibration curves to evaluate the predictive capability of the nomogram. The decision curve analysis was used to evaluate the net clinical benefits.

## 3 Results

### 3.1 Participants characteristics

**Figure 2:**
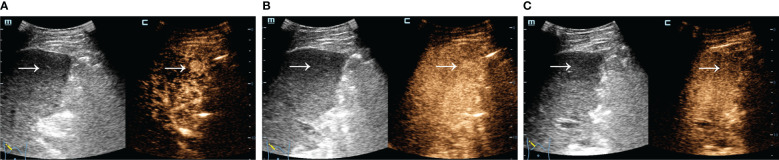
SCEUS image of a well differentiated HCC. Male, 61 years old, AFP=8.95 ng/ml. A slightly iso-echoic lesion of approximately 1.4*1.4 cm in size (arrow) with poorly defined boundary and regular morphology was seen in S5. It showed overall rapid and homogeneous hyper-enhancement in the AP **(A)**, iso-enhancement with no significant washout in the PVP **(B)**, and mild washout in the KP **(C)**. The final pathological diagnosis was well differentiated HCC.

**Figure 3:**
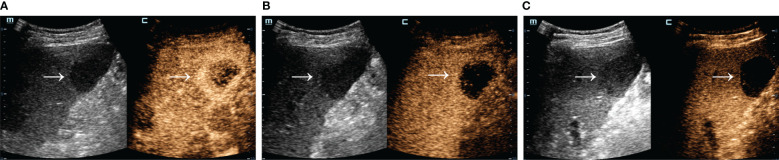
SCEUS image of a liver metastasis from pancreatic cancer. Male, 67 years old. A slightly hypo-echoic lesion of about 4.0*3.8 cm in size (arrow) with well-defined boundary and regular morphology was seen in S5. The AP **(A)** showed a rapid rim ring of hyper-enhancement, the PVP **(B)** showed hypo-enhancement, and the KP **(C)** was obviously washout. Definitive pathologic diagnosis was liver metastasis from pancreatic cancer.

A total of 324 patients with malignant focal liver lesions were enrolled in this study. After screening for study inclusion and exclusion criteria, 165 patients with AFPN-HCC were finally enrolled. The patient selection flowchart and grouping are shown in **Figure 1**. The baseline features of the subjects, grouped by AFPN-HCC and OM of the liver, were listed in **Table 1**. Among them, 85 (51.52%) were patients with AFPN-HCC, while 80 (48.48%) were patients with OM, including hepatic metastases (n=55), ICC (n=12), hepatic lymphomas (n=7), hepatic neuroendocrine tumor (n=4), primary malignant hepatic mesothelioma (n=2). The age of the subjects was (62.08 ± 10.28) years, and 110 (66.67%) were male. Subjects in the AFPN-HCC group were more likely to be male, and were noted to have higher BMI, alanine aminotransferase, total bilirubin and high-density lipoprotein, while triglyceride were lower. In addition, they were tended to have a single lesion without necrosis, and the SCEUS features were overall homogeneous enhancement in the AP, iso-enhancement in the PVP and DP, and mild/moderate washout in the KP. **Figure 2** demonstrates SCEUS image of an example of well differentiated HCC, while **Figure 3** shows a case of liver metastasis from pancreatic cancer.

**Table 1:** Characteristics of 165 patients with focal liver lesions.

Variables	Total (n = 165)	OM (n = 80)	AFPN-HCC (n = 85)	*P*
Age, years	62.08 ± 10.28	62.45 ± 9.66	61.73 ± 10.88	0.654
Gender				0.015
Female	55 (33.33)	34 (42.5)	21 (24.71)	
Male	110 (66.67)	46 (57.5)	64 (75.29)	
BMI, kg/m^2^	23.60 ± 3.54	22.98 ± 3.56	24.18 ± 3.44	0.029
Fasting plasma glucose, mmol/L	6.46 ± 2.57	6.86 ± 2.87	6.09 ± 2.20	0.055
Alanine aminotransferase, U/L	27.00 (17.00, 44.00)	21.00 (15.75, 41.00)	31.00 (19.00, 52.00)	0.017
Aspartate aminotransferase, U/L	30.00 (22.00, 50.00)	27.00 (19.75, 53.25)	33.00 (25.00, 50.00)	0.078
Albumin, g/L	37.68 ± 7.59	38.09 ± 9.02	37.28 ± 5.96	0.492
Total bilirubin, umol/L	15.70 (11.00, 26.60)	13.50 (9.38, 21.60)	17.60 (12.50, 27.60)	0.032
Gamma-glutamyl transferase, U/L	61.00 (29.00, 118.00)	63.00 (29.00, 138.00)	58.00 (35.00, 102.00)	0.222
Total cholesterol, mmol/L	4.37 ± 1.21	4.38 ± 1.18	4.35 ± 1.25	0.847
Triglyceride, mmol/L	1.23 (0.85, 1.87)	1.56 (0.93, 1.95)	1.11 (0.76, 1.72)	0.012
High-density lipoprotein, mmol/L	1.10 ± 0.34	1.04 ± 0.35	1.15 ± 0.32	0.04
Low-density lipoprotein, mmol/L	2.75 ± 0.89	2.77 ± 0.87	2.73 ± 0.92	0.77
Serum uric acid, umol/L	311.75 ± 127.87	294.14 ± 141.02	328.32 ± 112.46	0.086
Hypertension				0.501
No	89 (53.94)	41 (51.25)	48 (56.47)	
Yes	76 (46.06)	39 (48.75)	37 (43.53)	
Diabetes				0.054
No	109 (66.06)	47 (58.75)	62 (72.94)	
Yes	56 (33.94)	33 (41.25)	23 (27.06)	
Tumor location				0.525
Left lobe	45 (27.27)	20 (25.00)	25 (29.41)	
Right lobe	120 (72.73)	60 (75.00)	60 (70.59)	
Tumor echo				0.196
Hypo-echoic	109 (66.06)	50 (62.50)	59 (69.41)	
Iso-echoic	24 (14.55)	10 (12.50)	14 (16.47)	
Hyper-echoic	32 (19.39)	20 (25.00)	12 (14.12)	
Tumor size	4.23 ± 2.30	4.57 ± 2.63	3.91 ± 1.90	0.064
Tumor number				< 0.001
Single	82 (49.70)	23 (28.75)	59 (69.41)	
Multiple	83 (50.30)	57 (71.25)	26 (30.59)	
Boundary				0.418
Well defined	94 (56.97)	43 (53.75)	51 (60)	
Poorly defined	71 (43.03)	37 (46.25)	34 (40)	
Morphology				0.072
Regular	118 (71.52)	52 (65)	66 (77.65)	
Irregular	47 (28.48)	28 (35)	19 (22.35)	
Envelope				0.147
Without	90 (54.55)	39 (48.75)	51 (60)	
With	75 (45.45)	41 (51.25)	34 (40)	
Necrosis in tumor				< 0.001
Without	78 (47.27)	16 (20)	62 (72.94)	
With	87 (52.73)	64 (80)	23 (27.06)	
Arterial phase enhancement pattern				< 0.001
Rim	25 (15.15)	21 (26.25)	4 (4.71)	
Overall	140 (84.85)	59 (73.75)	81 (95.29)	
Arterial phase perfusion velocity				0.058
Quick enter	158 (95.76)	74 (92.5)	84 (98.82)	
Simultaneous enter	7 ( 4.24)	6 (7.5)	1 (1.18)	
Arterial phase enhanced level				0.486
Hyper-enhancement	152 (92.12)	73 (91.25)	79 (92.94)	
Iso-enhancement	11 ( 6.67)	5 (6.25)	6 (7.06)	
Hypo-enhancement	2 ( 1.21)	2 (2.5)	0 (0)	
Arterial phase enhanced homogeneity				< 0.001
Homogeneous	67 (40.61)	22 (27.5)	45 (52.94)	
Heterogeneous	98 (59.39)	58 (72.5)	40 (47.06)	
Arterial phase enhanced margin				0.079
Well defined	73 (44.24)	41 (51.25)	32 (37.65)	
Poorly defined	92 (55.76)	39 (48.75)	53 (62.35)	
Arterial phase tumor morphology				0.891
Regular	104 (63.03)	50 (62.5)	54 (63.53)	
Irregular	61 (36.97)	30 (37.5)	31 (36.47)	
Portal venous phase and delayed phase clearance velocity				0.875
Quick washout	127 (76.97)	62 (77.5)	65 (76.47)	
Simultaneous washout	38 (23.03)	18 (22.5)	20 (23.53)	
Portal venous phase and delay phase enhanced level				< 0.001
Hypo-enhancement	99 (60.00)	60 (75.00)	39 (45.88)	
Iso-enhancement	62 (37.58)	19 (23.75)	43 (50.59)	
Hyper-enhancement	4 ( 2.42)	1 (1.25)	3 (3.53)	
Kupffer phase enhanced level				0.063
Hypo-enhancement	147 (89.09)	75 (93.75)	72 (84.71)	
Iso-enhancement	18 (10.91)	5 (6.25)	13 (15.29)	
Kupffer phase degree of washout				< 0.001
Obvious	68 (41.21)	48 (60)	20 (23.53)	
Mild /Moderate	97 (58.79)	32 (40)	65 (76.47)	

### 3.2 Risk prediction nomogram development

**Figure 4:**
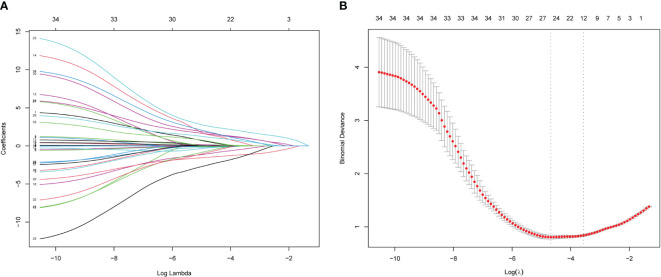
Features election using LASSO binary logistic regression model. **(A)** Log (lambda) value of 34 features in the LASSO model. A coefficient profile plot was produced against a log (lambda) sequence. **(B)** Parameter selection in the LASSO model uses five-fold cross-validation through minimum criterion. Partial likelihood deviation (binomial deviation) curves and logarithmic (lambda) curves are plotted. Minimum standard and 1-SE of the minimum standard are used to draw a vertical dashed line at the optimal value. Optimal lambda produces 12 nonzero coefficients. LASSO, least absolute shrinkage and selection operator.

Of the above characteristics, 12 were chosen according to the nonzero coefficients calculated through LASSO logistic regression analysis (**Figure 4**). The selected characteristics included BMI, albumin, gamma-glutamyl transferase, high-density lipoprotein, tumor echo, envelope, necrosis in tumor, AP enhancement pattern, quick entry in AP, PVP and DP enhanced level, KP degree of washout. These characteristics were then included in the multivariate logistic regression analysis.

**Figure 5:**
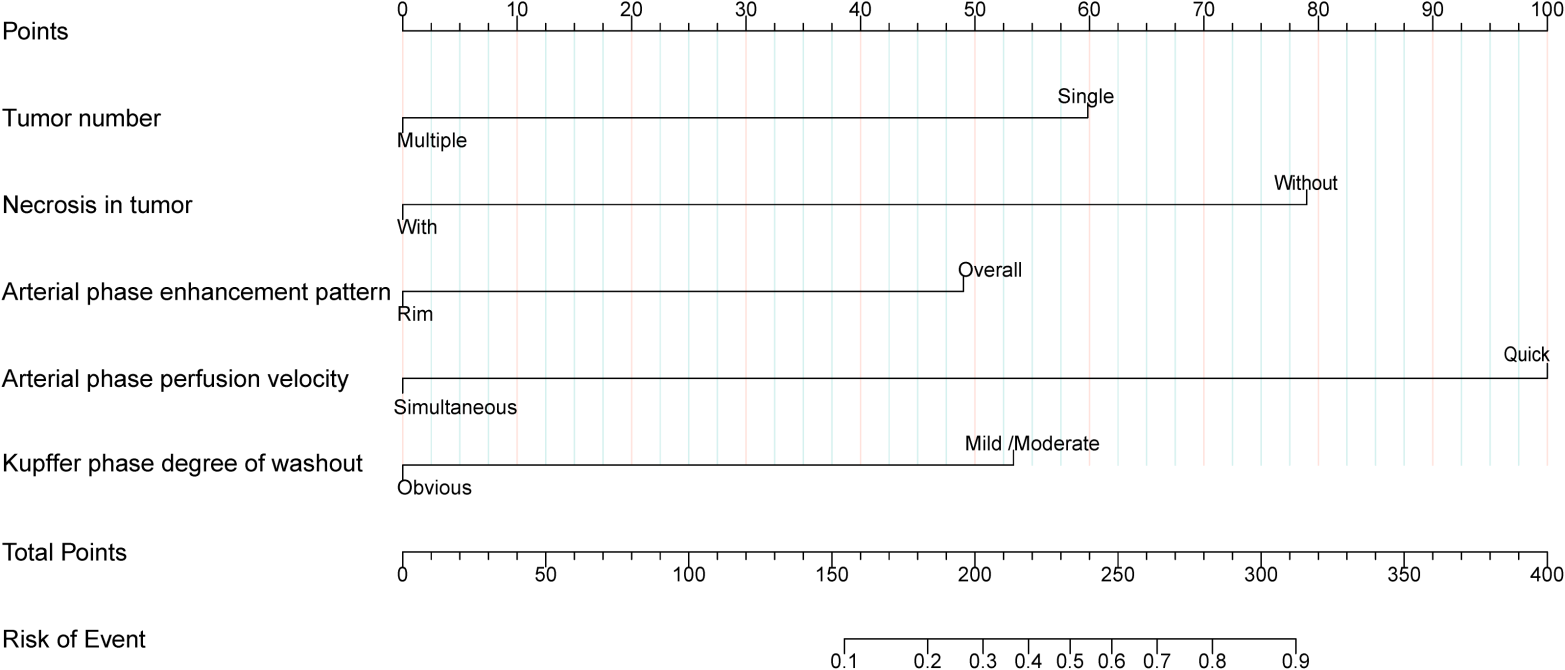
Nomogram for differentiating AFPN-HCC from OM. The first line represents the scoring scale. Corresponding scores for each predictor factor are shown in lines 2-6. The score for each predictor is determined by referencing the first line. The total score for the risk evaluation is the sum of each predictor score. To differentiate between AFPN-HCC and OM, the score point is located on the total points line (line 7). Then, the user descends vertically to the risk of complication (line 8).

Multivariate logistic regression analysis revealed that tumor number, necrosis in tumor, AP enhancement pattern, AP perfusion velocity and KP degree of washout were independent predictors of AFPN-HCC (**Table 2**). Therefore, we integrated these independent predictors to develop a predictive nomogram (**Figure 5**), with the higher score indicating the higher risk of AFPN-HCC.

**Table 2:** Logistic univariate and multivariate proportional hazard models of risk factors.

Variable	Univariate		Multivariate	
	OR (95CI%)	*P*	OR (95CI%)	*P*
BMI	1.105 (1.008~1.211)	0.0323	1.139 (0.988~1.313)	0.0726
Albumin	0.986 (0.946~1.027)	0.4908		
Gamma-glutamyl transferase	0.997 (0.994~1.000)	0.0433	0.997 (0.992~1.002)	0.2136
High-density lipoprotein	2.622 (1.037~6.630)	0.0416	2.503 (0.675~9.282)	0.1700
Tumor echo				
Hypoechoic	1(Ref)			
Isoechoic	1.186 (0.485~2.903)	0.7080		
hyperechoic	0.508 (0.226~1.142)	0.1012		
Tumor number	0.178 (0.091~0.347)	<0.001	0.209 (0.083~0.525)	0.0009
Envelope	0.634 (0.342~1.175)	0.1478		
Necrosis in tumor	0.106 (0.052~0.217)	<0.001	0.117 (0.046~0.2990)	<0.001
Arterial phase enhancement pattern	7.208 (2.35~22.105)	0.0006	4.669 (1.294~16.844)	0.0186
Quick entry in the arterial phase	0.147 (0.017~1.247)	0.0788	0.040 (0.003~0.586)	0.0187
Portal venous phase and delay phase enhanced level				
Hypo-enhancement	1(Ref)		1(Ref)	
Iso-enhancement	3.482 (1.775~6.830)	0.0003	2.545 (0.909~7.127)	0.0754
Hyper-enhancement	4.615 (0.463~45.981)	0.1922	17.619 (0.462~671.417)	0.1224
Kupffer phase degree of washout	4.875 (2.490~9.544)	<0.001	3.104 (1.152~8.362)	0.0251

### 3.3 Performance and validation of the nomogram

**Figure 6:**
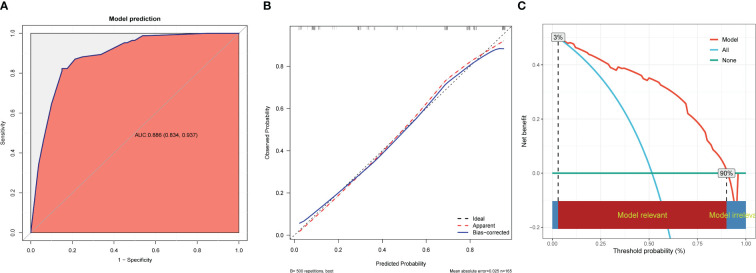
Performance and validation of the nomogram. **(A)** ROC curve of the nomogram. The point on the curve represents the optimal cutoff value (specificity, sensitivity). The brackets next to the area under the ROC curve (AUC) represent the 95% confidence interval. **(B)** Calibration curve of the nomogram. The apparent curve represents the relationship between predicted and actual probabilities of clinically significant complications. The bias-corrected curve is plotted by bootstrapping using 1,000 resamples. The ideal curve is the 45° line, which indicates perfect prediction. **(C)** Decision curve analysis of the nomogram. Red solid lines represent the nomogram, x axis, cutoff probability, and y axis, net benefit. AUC, area under the curve; ROC, receiver operating characteristic.

The receiver operating characteristic curve showed that the nomogram had an AUC of 0.886 (95% CI, 0.834-0.937) (**Figure 6A**). The best predictive value was 0.524, with a sensitivity of 82.35%, specificity of 85.00%, positive predictive value of 85.37%, negative predictive value of 81.93%, and accuracy of 83.64%. Furthermore, the Hosmer-Lemeshow test showed a good fit (*P*=0.592). The calibration curve showed high agreement between the predicted and the actual results when internal validation was performed using 1,000 bootstrap samples (**Figure 6B**), and the AUC of internal validation was 0.865. An assessment of the decision curve analysis of the clinical utility of the nomogram showed that, with 3%–90% probabilities, the application of the nomogram resulted in a more significant net benefit in comparison with the treat-all or treat-none strategies (**Figure 6C**).

## 4 Discussion

AFP combination with imaging examinations such as ultrasound is an important means for clinical screening and diagnosis of HCC, but about one-third of HCC patients are AFP-negative, which poses challenges in clinical diagnosis and differentiation (5). The treatment options and clinical prognosis of AFPN-HCC are different from OM, thus an accurate differentiation of AFPN-HCC is helpful in developing clinical treatment strategies and improving patient prognosis.

In this study, we developed and validated a predictive model for preoperative noninvasive differential diagnosis of AFPN-HCC and OM, which was based on conventional ultrasound and SCEUS features, including tumor number, necrosis in tumor, AP enhancement pattern, AP perfusion velocity, and KP degree of washout. We used an integrated analytical methodology including LASSO, univariate and multivariate logistic regression analyses. The model showed good diagnostic ability in separating AFPN-HCC from OM, with the AUC, sensitivity and specificity of 0.886, 82.35% and 85.00%, respectively. The calibration curve indicated that the predicted results were in high agreement with the actual results when 1,000 bootstrap samples were used for internal validation. It demonstrated the necessity of its application in timely identification of AFPN-HCC. Compared with previous studies, which mainly relied on CT or MRI image characteristics, our study is unique in using features in SCEUS for modeling for distinguishing AFPN-HCC from OM (9–11).

As the new generation of liver-specific ultrasound contrast agent, Sonazoid has an effective imaging time of more than 2 hours, providing more adequate time for clinical examination and treatment. Besides, Sonazoid can be engulfed by hepatic Kupffer cells, forming specific imaging of the post-vascular phase (12–14). The KP, as the post-vascular phase, was obtained with a delay of more than 10 min after injection. It has been demonstrated that the SCEUS feature of KP washout maintains a relatively balanced sensitivity and specificity (15). Due to the lack of normal liver tissue and Kupffer cells, hepatic malignant tumors show hypo-enhancement in the KP, whereas benign lesions maintain iso-enhancement, which significantly improves the sensitivity and specificity of the identification of benign and malignant liver focal lesions (16, 17).

Few studies have focused on the differentiation of AFPN-HCC from OM using SCEUS. A meta-analysis was performed to evaluate the accuracy of CEUS in differentiating malignant from benign FLLs, showed that Sonazoid had the highest diagnostic accuracy among three major contrast agents (18). Ohama H et al. enrolled 73 patients with HCC in a study to compare the post-vascular phase of SCEUS with the hepatobiliary phase of gadolinium ethoxybenzyl diethylenetriamine (Gd-EOB-DTPA) of MRI, confirmed that the hypo-echoic presentation of the KP may be specific to HCC, especially in progressive HCC (19). Sugimoto K et al. included 78 HCC and dysplastic nodules, suggested that KP may be useful in estimating the histologic grade, especially in moderately and poorly differentiated types (20). A multi-center study conducted by Wang S et al. involved 41 cases of ICC and 49 cases of poorly differentiated HCC, and established a predictive model for poorly differentiated HCC and ICC based on SCEUS and clinical characteristics (21). Due to the high similarity between AFPN-HCC and some OM (like ICC and hepatic metastatic carcinoma) in conventional ultrasound, there were challenges in differential diagnosis between them.

Our results showed that the majority of AFPN-HCC were single, hypo-echoic, and rarely with internal necrosis; SCEUS showed overall, rapid and homogeneous hyper-enhancement in the AP; iso- or hypo-enhancement in the PVP and DP; and mild washout with hypo-enhancement in the KP. These findings were consistent with Wang et al. (22). It is mainly due to that AFPN-HCC is supplied by hepatic artery or by hepatic artery and portal vein, thus it shows hyper-enhancement in the AP, which is consistent with typical HCC, ICC or OM. Several studies showed that AFPN-HCC were mostly well-differentiated HCC with less necrosis and may certain an amount of Kupffer cells, thus the difference between AFPN-HCC and OM lies in the mild washout in the KP, showing iso- or hypo-enhancement, which was consistent with our findings (20, 23). In tumors, the phagocytic function of macrophages is in part associated with tumor progression. In addition, most of the OMs were multiple, large, hypo-echoic, and accompanied with necrosis internally; SCEUS showed heterogeneous hyper-enhancement in the AP by rim-enhancement; rapid washout with hypo-enhancement in the PVP and DP; and obvious washout with hypo-enhancement in the KP. A meta-analysis has proved that rim hyper-enhancement in the AP could be applied for detecting non-HCC malignant tumors (24). Rim hyper-enhancement in the AP was more commonly seen in metastases (25–27), suggesting peripheral neovascularization and central necrosis, together with the elevated levels of reactive cellular components in the tumor margins and the surrounding noncancerous tissues, which disappeared in the PVP. Since the OMs have almost no Kupffer cells within it, it was obviously washout in the KP, as mentioned in the research of Li L et al. (28).

Our study also has some limitations. First of all, the data were obtained from a single-center, which may lead to a degree of confounding bias, and our conclusions should be validated by prospective multicenter researches in the future. What’s more, the sample size of our study is relatively small that future studies should prospectively collect more data from multicenters to verify the nomogram externally and improve its validity. Furthermore, we used 10 EPV for sample size calculation and did not consider the event rate. In addition, we did not analyze the capability of CEUS LI-RADS features to distinguish AFPN-HCC from OM, which should be further investigated in the future. We will continue to gather relevant data to establish diagnostic models for intrahepatic malignant tumors, which can better guide the clinical practice for individualized treatment.

## 5 Conclusion

This study found that tumor number, necrosis in tumor, AP enhancement pattern, AP perfusion velocity, and KP degree of washout contribute to the diagnosis of AFPN-HCC. Based on these characteristics, the nomogram has the potential to provide a non-invasive diagnosis of AFPN-HCC preoperatively, which can offer some support for clinical individualized treatment decisions.

## Data Availability

The datasets used and analyzed during the current study are available from the corresponding author on reasonable request. Requests to access the datasets should be directed to liuliping1600@sina.com.
