# Supplementary material for: Differentiation of AFP-negative hepatocellular carcinoma from other intrahepatic malignant lesions by a noninvasive predictive model based on Sonazoid contrast-enhanced ultrasound
Source: Front Oncol. 2025 Jul 17;15:1623670. doi: 10.3389/fonc.2025.1623670 (PMC12312008; doi:10.3389/fonc.2025.1623670)
Supplement: Supplementary file 1 [file Table1.docx]

**Supplementary Table 1. Inter-observer variability analysis.**

| Variables | Doctor A | Doctor B | κ | *P* |
| --- | --- | --- | --- | --- |
| Necrosis in tumor |  |  | 0.939 | <0.001 |
| Without | 77 | 1 |  |  |
| With | 4 | 83 |  |  |
| Arterial phase enhancement pattern | |  | 0.906 | <0.001 |
| Rim | 23 | 2 |  |  |
| Overall | 2 | 138 |  |  |
| Arterial phase perfusion velocity | | | 0.827 | <0.001 |
| Quick enter | 158 | 0 |  |  |
| Simultaneous enter | 2 | 5 |  |  |
| Arterial phase enhanced level | |  | 0.833 | <0.001 |
| Hyper-enhancement | 150 | 2 |  |  |
| Iso- or hypo-enhancement | 2 | 11 |  |  |
| Arterial phase enhanced homogeneity | |  | 0.913 | < 0.001 |
| Homogeneous | 65 | 2 |  |  |
| Heterogeneous | 5 | 93 |  |  |
| Arterial phase enhanced margin | |  | 0.890 | < 0.001 |
| Well defined | 71 | 2 |  |  |
| Poorly defined | 7 | 85 |  |  |
| Arterial phase tumor morphology | |  | 0.898 | < 0.001 |
| Regular | 97 | 7 |  |  |
| Irregular | 1 | 60 |  |  |
| Portal venous phase and delayed phase clearance velocity | | | 0.915 | < 0.001 |
| Quick washout | 124 | 3 |  |  |
| Simultaneous washout | 2 | 36 |  |  |
| Portal venous phase and delay phase enhanced level | | | 0.923 | < 0.001 |
| Hypo-enhancement | 99 | 0 |  |  |
| Iso- or hyper-enhancement | 6 | 60 |  |  |
| Kupffer phase enhanced level | |  | 0.813 | <0.001 |
| Hypo-enhancement | 144 | 3 |  |  |
| Iso-enhancement | 3 | 15 |  |  |
| Kupffer phase degree of washout | | | 0.950 | <0.001 |
| Obvious | 64 | 4 |  |  |
| Mild /Moderate | 0 | 97 |  |  |
